# Association of molds and metrological parameters to frequency of severe asthma exacerbation

**DOI:** 10.1186/s13223-019-0323-8

**Published:** 2019-04-29

**Authors:** Mona Al-Ahmad, Edin Jusufovic, Nermina Arifhodzic, Tito Rodriguez, Jasmina Nurkic

**Affiliations:** 1 Microbiology Department, Faculty of Medicine, Kuwait University, P.O. Box 24923, 13110 Safat, Kuwait; 2 Al-Rashed Allergy Center, Ministry of Health, Kuwait City, State of Kuwait; 3 Medical Faculty, University of Tuzla, Tuzla, Bosnia and Herzegovina

**Keywords:** Asthma exacerbation, Molds, Meteorological parameters

## Abstract

**Background:**

Sensitization to airborne molds may be a risk factor for severe asthma and direct cause of asthma exacerbation (AE).

**Methods:**

A prospective, 1-year (April 2016–March 2017) study, done in Kuwait Allergy Centre, investigated the link between AEs with exposure to outdoor molds and the role of meteorological parameters in mold sensitized patients and compared with non-allergic asthma patients who had asthma deterioration. The total of 676 adult asthmatics with moderate-severe AEs were included and divided into atopic (85.65%) and non-atopic group. Atopy was defined by positive skin prick test (SPT) to at least one inhalant allergen. Data regarding atopy and asthma severity were collected from patient’s records. Patients with symptoms and signs of acute respiratory infection and patient sensitized to indoor allergens only were excluded. Daily count of local pollens (Salsola kali, Bermuda grass) and molds (Aspergillus, Alternaria and Cladosporium) were obtained from the Aerobiology department. Daily metrological parameters (atmospheric pressure-AP, temperature-T and relative humidity-RH) were provided by Kuwait Environment Public Authority. Count of spores/m^3^ and weather variable are shown on weekly basis. The year circle was divided into 4 Seasons (1, 2, 3, 4) accordingly to typical desert climate.

**Results:**

Sensitization to molds was relatively high but significantly less (25.0%) if compared to the pollens sensitization. The highest number of AEs was in season 4 for both molds and pollens sensitized patients. Seasonal patterns for both allergens were significant and positively correlated with RH and AP. In season 1 only, mold sensitized patients showed higher rate of AEs. Non-atopic patients have been less sensitive to increased RH than atopic. Negative correlation with T was similar in both atopic and non-atopic patients.

**Conclusion:**

Despite of high rate of sensitization to molds, their significant role in triggering AE was not found in desert environment. Typical desert climate and high allergencity of local weeds outweigh the influence of the molds.

## Introduction

There is robust evidence of close relationship between elevated environmental fungal spore count in the atmosphere and asthma deterioration [10, 63]. Exposure to mold allergens in mold sensitized patients is considered as a strong risk factor for asthma severity and occurrence of asthma exacerbations as well [11, 50]. The most common mold allergen sources belong to the taxonomic group Fungi Imperfecti of Ascomycetes, which include Alternaria, Cladosporium and Aspergillus species. Their allergens have been best characterized [8, 60, 66] and approved as a risk factor for the development of more severe asthma [50, 55], occurrence of epidemic and thunderstorm related AEs [10, 12, 15, 18, 19, 36, 37, 47, 50] and might increase asthma mortality [11]. It has been shown that exposure to the higher concentration of Alternaria, Aspergillus, Cladosporium and Penicillium species increases number of AE by 36% to 48% compared to those exposed to lower level of those fungi [13].

Molds are ubiquitous, growing anywhere if there is a source of worm and moisture. Alternaria and Cladosporium are common in outdoor environment while Aspergillus species predominate indoors [29, 31]. Exposure and sensitization of susceptible people to outdoor molds is more relevant [9, 10, 46] than indoor. Outdoor fungal aerosol is primarily derived from their growth on plants [25]. Their concentration highly depends on environmental factors and climate conditions [23, 48, 64]. Thus, mold concentration is higher in the late summer and autumn, caused by biomaterial degradation. The aerodynamic diameter of allergenic molds is small (2–10 pm). Hence, mold spores readily penetrate into the lower airways [8, 62] where they cause symptoms in susceptible people.

Diagnosis of mold allergy depends on the demonstration of mold-specific IgE, either by skin testing or in vitro tests [26]. However, due to the poor quality of fungal allergen extracts, diagnosis of fungal allergy is hampered [27, 62, 64]. In order to confirm the direct link of particular AE and molds is rather difficult. In addition, frequent co-existence of sensitization to other inhalant allergens, as well as the absence of reliable typical clinical history which confirm the exclusive mold exposure as culprit, make this issue more difficult.

The estimated sensitization prevalence to mold with commercial, still poorly standardized allergen extracts [27] range from 3 to 10% [64], while in allergic asthma patients vary from 11 to 14.8% (Hasnain et al. [24]), [5] to more than 30% [3, 62, 64, 67]. Molds as a prominent source of allergens are still neglected in both basic research and clinical practice [10].

Data regarding association of AE with mold sensitization are limited in the Middle East.

The primary objective of this study was to assess the influence of outdoor molds on the occurrence of AE in adult atopic asthma patients sensitized to molds and compare it with AE in non-atopic asthmatics.

The secondary objectives were to determine effect of meteorological factors on molds concentration in desert environment and their relation with AEs.

## Patients and methods

Retrospective study was done at the Al Rashed Allergy Centre in Kuwait and was funded by research grant from Kuwait University (research project No: ZM02/16). Ethical clearance was granted by Kuwait University as well.

A total 676 asthmatic patients (> 15 years old) in mild-moderate-severe AE were included and divided into: atopic, sensitized to outdoor allergens (pollen and molds) (n = 579; 85.65%) and non-atopic (n = 97; 14.35%). Atopy was defined by a positive skin prick test (SPT) to at least one inhalant commercial allergen extract (Diater, Spain), which include molds (Aspergillus, (1:20w/v), Alternaria (1:20w/v) and Cladosporium 1:40w/v.), and local pollens (Bermuda grass and Salsola kali weed). A normal saline and histamine (10 mg/ml) have been used as negative and positive control. A SPT wheal size diameter > 3 mm was an evidence for specific allergen sensitization. AEs were recorded through the whole year circle (April 2016–March 2017 year).

Moderate-severe AE was defined accordingly to the clinical and management criteria proposed consensus [38] applicable for clinical trials by The American Thoracic Society (ATS), European Respiratory Society (ERS) and World Allergy Organization (WAO) as follow: (1) use of systemic corticosteroids or an increase from a stable maintenance dose, for at least 3 days; (2) courses of corticosteroids separated by 1 week, were considered as separate severe AE; (3) hospitalization or emergency room visit because of asthma, requiring systemic corticosteroids.

Patients sensitized to indoor allergens only (dust mites and animal dander) and those with a presence of acute respiratory tract infection, confirmed clinically with signs and symptoms and positive laboratory parameters suggestive of acute inflammation (elevated C-reactive protein and neutrophil counts), were excluded from the study.

Daily atmospheric spore count of pollens and molds (Aspergillus, Alternaria and Cladosporium) were collected by Burkard 7-day volumetric spore trap. Burkard traps were fixed at several monitoring stations which covered all inhabited urban areas of the country, they were positioned in relation to where their participants resided.

Concentrations of pollens and molds were obtained from the Aerobiology department in the Al Rashed Allergy Centre and presented as a mean weekly count.

Due to well-known seasonal fluctuation of outdoor molds concentration, the year circle was divided into 4 seasons (1, 2, 3, 4) according to typical weather characteristics for the region where study was performed: Season 1: December–February, Season 2: March–May, Season 3: June–August and Season 4: September–November.

Daily meteorological parameters of relative humidity (RH) temperature (T) and atmospheric pressure (AP) were obtained from Kuwait Environment Public Authority and also presented as a mean weekly count.

### Statistical analysis

Non-parametric and parametric methods are used to calculate statistical significance. The distribution value is determined D’Agostino and Pearson omnibus test normality. Student’s t-test, Mann–Whitney test, Fisher’s test and χ^2^ test were used for calculating the difference between the groups. ANOVA test was used to calculate the relative difference distribution variance between variables. The statistical hypotheses were tested at the level of α = 0.05, and the difference between the groups in the sample was considered significant when *p *< 0.05 or less. Statistical significance was depicted as: *p *< 0.05, *p *< 0.01 and *p *< 0.001. All data were analysed using GraphPad Prism version 7 (San Diego, California, USA).

## Results

Study included 676 patients with AE (mean age: 47.15 ± 14.66 years; female/male ratio: 0.77).

Females were significantly older than male patients (*p *< 0.01). No gender difference in distribution of asthma severity (female vs. male: 0.36/81.59/18.05 vs. 0.26/82.51/17.23) and between two groups (atopic vs. non-atopic 0.35/81.38/18.26 vs. 0.0/86.46/23.54; *p *> 0.05 for both measurements). Presence of nasal symptoms was more common in female patients (*p *< 0.05) (Table 1).

**Table 1:** Basic characteristics of patients

	Female	Male	*p* value
Number (n; %)	294 (43.49%)	382 (56.51%)	–
Mean age (years)	49.28 ± 0.9	45.97 ± 0.75	0.005*
Asthma severity (mild/moderate/severe)	0.36/81.59/18.05	0.26/82.51/17.23	0.937
Nasal symptoms (%)	79.23	71.37	0.055

	Atopic	Non atopic	*p* value
Number (n; %)	579 (85.65%)	97 (14.35%)	–
Gender F/M (%)	43.95/56.05	41.24/58.76	0.658
Mean age (years)	47.21 ± 14.89	49.30 ± 13.12	0.209
Asthma severity (mild/moderate/severe)	0.35/81.38/18.26	0.0/86.46/13.54	0.382
Nasal symptoms (%)	72.15	58.51	0.102

Outdoor allergens	Salsola kali	Bermuda grass	Total pollens	Alternaria	Aspergillus	Cladosporium	Total moulds
Sensitization (n; %)	356 (61.48%)	198 (34.2)	421 (72.71)	95 (16.41)	60 (10.36)	56 (9.67)	145 (25.04)
Mono-sensitization (n; %)	146 (25.21)	25 (4.32)	171 (29.53%)	8 (1.38)	0 (0.0)	0 (0.0)	8 (1.38)

* Difference was significant

According to positivity of skin prick tests (SPT) majority of patients were atopic (n = 579; 85.65%) with atopic vs. non-atopic ratio of 5.97 (Table 1).

Sensitization to outdoor molds was less frequent (145/25.04%) in comparison to local pollens of weed, Salsola kali and Bermuda grass (421/72.71%) (*p *< 0.0001 for both measurements). Sensitization to Alternaria was more frequent than sensitization to Aspergillus and Cladosporium (*p *< 0.01 and *p *< 0.001). Small number of patients (1.8%) has been sensitized to one mold species (Alternaria), while sensitization to Cladosporium or Aspergillus only was not observed (Table 1).

Average concentration of local pollens was higher in Season 4 compared to Seasons 1, 2 and Season 3 (*p *< 0.0001 for all measurements). Concentration of pollen was lower in Season 2 compared to Season 1 and 3 (*p *< 0.05 and *p *< 0.0001 respectively) and similar in Seasons 1 and 3 (*p *> 0.05).

Average concentration of Alternaria and Cladosporium was higher in Season 4 in comparison to Seasons 1, 2 and Season 3 (*p *< 0.0001 for all measurements). Concentration of Alternaria was higher in Season 3 compared to Season 1 (*p *< 0.05), but lower compared to Season 2 (*p *< 0.0001) and higher in Season 2 compared to Season 1 (*p *< 0.05). Number of Cladosporium spores was also higher in Season 2 if compared to Seasons 1 and 3 (*p *< 0.0001 both measurements), as well as in Season 1 compared to Season 3 (*p *< 0.0001).

Aspergillus species outdoor was detected in the lowest concentration, showing higher average concentration in Season 1 in comparison to Seasons 2, 3 and 4 (*p *< 0.01, *p *< 0.001 and *p *< 0.0001 respectively).

Average humidity was higher in Season 1 compared to other Seasons (*p *< 0.0001 for all measurements), although in Season 2 and 4 maximal humidity in particular days was high (78% and 72%, respectively) in contrast to three summer months (Season 3) when maximal relative humidity was 33%.

Average temperature (40 °C) was highest during Season 3, if compared with Season 1 (15 °C), 2 (25 °C) and 4 (30 °C) (*p *< 0.0001 for all measurements). Also, temperature was higher during Season 4 in comparison to Season 1 (*p *< 0.0001) and 2 (*p *< 0.01), as well as during Season 2 compared to Season 1 (*p *< 0.0001).

Average atmospheric pressure was higher in Season 1 (1020) compared to Seasons 2, 3 and Season 4 (*p *< 0.0001 for all measurements). The lowest atmospheric pressure was in Season 3 (1000) (Table 2).

**Table 2:** Average concentration of pollen (both Salsola kali and Bermuda grass), Alternaria, Aspergillus and Cladosporium in the air, as well as average relative humidity, temperature and atmospheric pressure per seasons

	Season 1	Season 2	Season 3	Season 4
Pollen	27.36 [0, 99.0]	20.16 [2.88, 44.64]	28.59 [16, 66.65]	840 [96.48, 1200]
Alternaria	37 [12.5, 580]	93 [14.6, 202.21]	55.33 [32.7, 101]	550 [40, 810]
Aspergillus	4.32 [0, 234.7]	7.61 [1.64, 27.15]	4.32 [0, 33.12]	0 [0, 30]
Cladosporium	206.7 [73.4, 1422.5]	423.57 [143.8, 1422.5]	129.6 [53.28, 771.84]	740 [194.4, 887]
Humidity (%)	40 [18, 88]	28 [9, 78]	12 [6, 33]	18 [9, 72]
Temperature (°C)	15 [6, 22]	25 [19, 39]	40 [30, 47]	30 [15, 45]
Atmospheric pressure (mmHg)	1020 [1013, 1026]	1013 [1005, 1019]	1000 [996, 1009]	1013 [999, 1023]

Data were shown as medians with minimum and maximum value

**Fig. 1:**
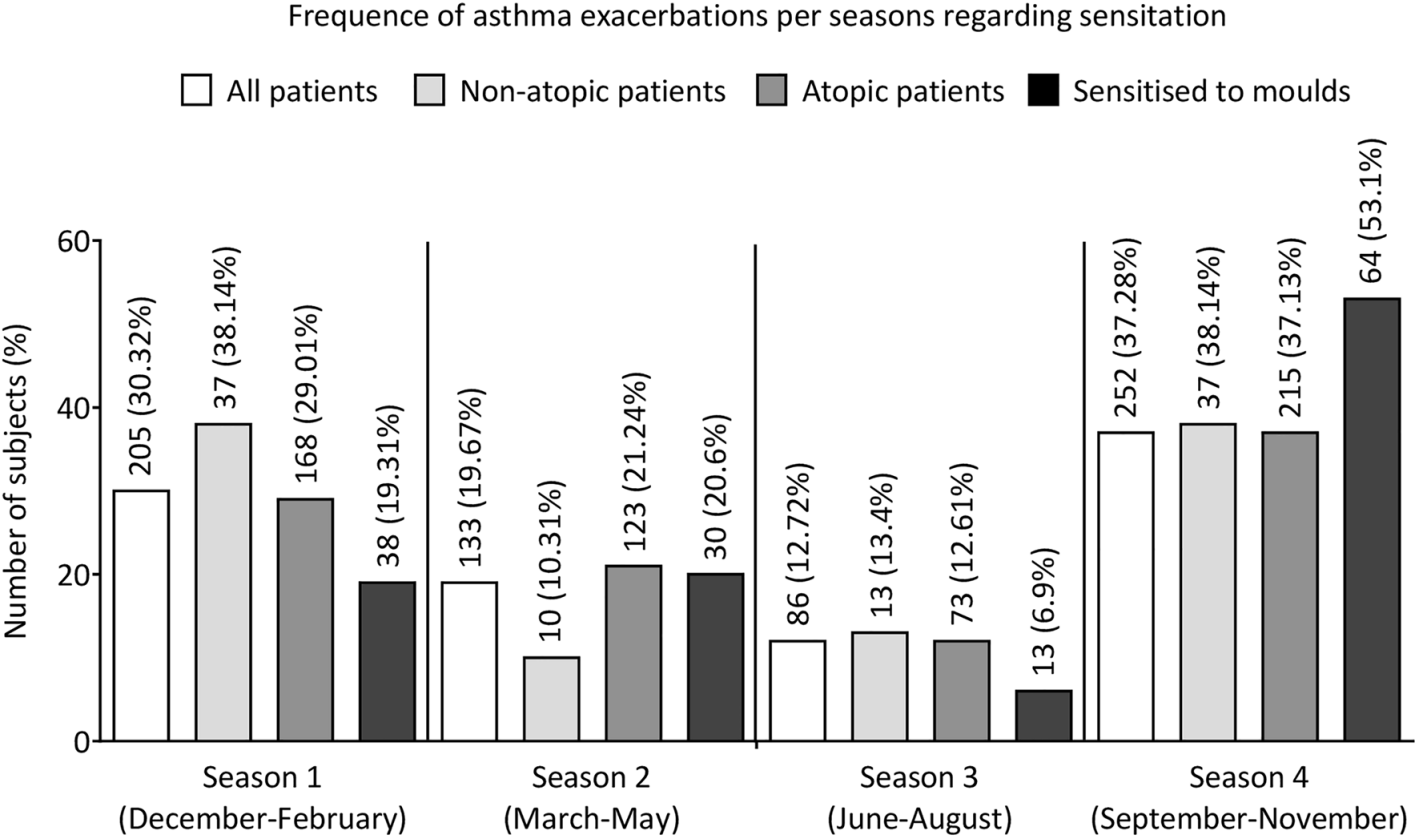
Shows AE per seasons in all patients and in regard to molds sensitization

In all patients more AE were observed in Season 4 than in Season 1, 2 and 3 (*p *= 0.0081, *p *< 0.0001 and *p *< 0.0001, respectively) (Fig. 1), more during Season 1 compared to Season 2 and 3 (*p *< 0.0001 for both measurements), as well as in Season 2 in comparison to Season 3 (*p *= 0.0007) (Fig. 1).

In non-atopic patients, more AE were observed in Seasons 1 and 4 than in Seasons 2 and 3 (*p *< 0.0001 for all measurements), while in atopic patients, more frequently AE were observed in Season 4 than in Season 1, 2 and 3 (*p *< 0.01, *p *< 0.001 and *p *< 0.0001, respectively) (Fig. 1).

Also in atopic patients more AE were observed in Season 1 compared to Season 2 and 3 (*p *< 0.01 and *p *< 0.0001). No difference was observed between two groups of patients in Season 1 and 4 (*p *> 0.05), and Seasons 2 and 3 (*p *> 0.05) (Fig. 1).

In patients sensitized to molds (25.4%) more patients had AE during Season 4 than in Season 1, 2 and 3 (*p *< 0.01, *p *< 0.01 and *p *< 0.001 respectively). In these patients, the least number of AE were observed during Season 3 in comparison to Season 1 and Season 2 (*p *< 0.001 for both measurements), but similar frequencies were observed between Season 1 and 2 (*p *> 0.05) (Fig. 1).

In non-atopic group, AE positively correlate to atmospheric pressure, but negatively to temperature (*p *< 0.05 for both measurements) (Table 3).

**Table 3:** Correlation between number of AE in non-atopic and atopic patients with concentrations of Aspergillus, Alternaria, Cladosporium and pollens (both Salsola kali and Bermuda grass), as well as with temperature, relative humidity and atmospheric pressure

	Number of AE Non-atopic patients			Number of AE Atopic patients		
	r	95% CI	*p* value	r	95% CI	*p* value
Aspergillus	0.5649	− 0.01354 to 0.8600	0.0278*	0.633	0.09268 to 0.8853	0.014*
Alternaria	0.7416	0.2918 to 0.9228	0.0029*	0.5575	− 0.02425 to 0.8572	0.03*
Cladosporium	0.5733	− 0.001017 to 0.8632	0.0257*	0.5906	0.02515 to 0.8698	0.022*
Pollens	0.8786	0.6145 to 0.9656	< 0.0001*	0.7013	0.2131 to 0.9093	0.005*
Temperature	− 0.4989	− 0.8340 to 0.1053	0.0494*	− 0.6194	− 0.8804 to − 0.07036	0.016*
Relative humidity	0.305	− 0.4524 to 0.6746	0.1642	0.5348	− 0.05661 to 0.8484	0.037*
Atmospheric pressure	0.5032	− 0.09953 to 0.8358	0.0477*	0.5429	− 0.04519 to 0.8515	0.034*

* Difference was statistically significant

Number of AE in atopic patients positively correlated with the increment of level of all three mold species (Aspergillus, Alternaria, Cladosporium) (*p *< 0.05, *p *< 0.05, *p *< 0.01 respectively), pollens (*p *< 0.001), relative humidity (*p *< 0.05) and atmospheric pressure (p < 0.05), but negatively with temperature (*p *< 0.05) (Table 3).

Number of AE in mold sensitized patients showed positive correlation to concentration of Cladosporium (*p *< 0.01), concentration of pollens’ (*p *< 0.001), relative humidity (*p *< 0.05) and atmospheric pressure (*p *< 0.05), but negative correlation (*p *< 0.05) with temperature. No significant correlation was found toward concentration of Aspergillus and Alternaria (*p *> 0.05 for both measurements) (Table 4).

**Table 4:** Correlation between numbers of AE in patient’s sensitized to molds with concentrations of Aspergillus, Alternaria, Cladosporium and pollens (both Salsola kali and Bermuda grass), as well as with temperature, relative humidity and atmospheric pressure

	Number of AE in patients sensitized to molds		
	R	95% CI	*p* value
Aspergillus	− 0.1131	− 0.6452 to 0.4929	0.726
Alternaria	− 0.1794	− 0.6831 to 0.4399	0.288
Cladosporium	0.7087	0.2271 to 0.9118	0.005*
Pollens	0.8402	0.5142 to 0.9541	0.0006*
Temperature	− 0.5138	− 0.8400 to 0.08543	0.044*
Relative humidity	0.5896	0.02357 to 0.8694	0.022*
Atmospheric pressure	0.5639	− 0.01493 to 0.8597	0.028*

* Difference was statistically significant

## Discussion

We investigated the link between AE in asthmatic patients sensitized to molds and pollens in a desert environment with the number of spores in the atmosphere, the influence of weather parameters on the molds and pollens’ concentration and compared that with a group of non-allergic asthmatics who had asthma deterioration. Although there is lots of evidence that exposure to molds might trigger asthma symptoms in mold-sensitized patients [4, 10, 15, 47, 50], mold production, stability variation and their enormous diversity under different condition causes limitation in tracking direct molds effect on asthma patients [47, 55, 62, 63]. O’Driscoll and collaborates [43] documented that 76% of patients with multiple admissions had positive skin test to at least one mold species compared with 16–19% patients who were SPT negative (*p *< 0.001). Contrary to that report, our results did not find the difference in the frequency of AEs between allergic and non-allergic asthma patients during the whole year circle. We speculated that low concentrations of outdoor molds in the desert might influence on such results (Table 1). Furthermore, the majority of our patients in both groups are diagnosed as having moderate asthma (0.35%/81.38%/18.26% vs. 0.0%/86.46%/13.54%; *p *< 0.05 for measurements) for all degree of severity (Table 1). Similarly, Kennedy and collaborates [32] have documented, although less frequently, the occurrence of asthma attacks in mold sensitized patients, who generally have only mild or moderate asthma [32]. Co-existence of nasal symptoms was common in both groups and was more frequent in male patients (Table 1).

Our results have shown a relatively high rate of sensitization to molds (25.4%), similar to the results found by others in the region, from 13% [1] to more than 40% [20, 23]. Regardless of that, a direct link between AEs and sensitization to molds was not confirmed. Furthermore, we rarely observed sensitization to a single mold species (1.38%), Eight of our patients have only been sensitized to Alternaria, while mono-sensitization to Cladosporium and Aspergillus was not observed (Table 1). This observation is likely related to extensive cross-link among different molds species. Similar observations are reported both in the region (3–4%) [5, 22] and in other climate conditions as well [27, 28].

The seasonal variation of outdoor molds concentration is an important pattern. In a temperate and moister climate the number of mold spores might be very high. It is documented that number of Alternaria spores might reach 50,000/m^3^ in summer/early fall [14]. Other studies [46] observed a significant increase of 1.39 of maximal asthma symptoms per 2 weeks for each tenfold increase in outdoor Alternaria. The same was also shown for other fungal species (Sttenet and Beggs [57]), [34, 61].

In the harsh desert climate, concentrations of molds also exhibit seasonal variation, being low during extremely hot, long and dry summer (Season 3) (maximal mean temperature exceed + 42 °C). Such weather conditions are unsuitable for molds to thrive and disperse. In that period of the year the lowest number of AE is observed (Table 1), while the highest number was found in Season 4 (September–November), being significantly higher than in Seasons 1, 2 and 3 (*p *< 0.01, *p *< 0.001 and *p *< 0.0001, respectively) (Fig. 1). Season 4 in the desert environment is characterized by slow reduction in outdoor temperatures, increase in relative humidity and increased number of mold spores in the air. In the same season however, the number of pollen count, especially weed pollen (Salsola kali/Chenopodiaceae family) was the highest (Fig. 1 and Table 1). That means the number of AE tracks both molds and pollens count (Fig. 1). The typical shape of pollen spores count we observed in the previous studies [7, 54], conducted in Kuwait. The obtained results in this study showed that sensitization to local pollens over-count all other inhalant allergens including molds (Table 1). This is consistent with results reported earlier from the same centre [2]. Similarly, recent meta-analysis regional results [41] documented the high rate of sensitization to local pollens (47%), being responsible for the high prevalence of respiratory allergy in the region. They concluded that sensitization to Alternaria and other non-regional aeroallergens only have a minor influence on asthma patients. It is in accordance with the results obtained in the present study, which document a significantly higher rate of sensitization to pollens in comparison to the rate of sensitization to molds (421/72% vs. 113/25.02%, respectively).

On the other hand, Pulimood and collaborates in 2007 documented that Alternaria sensitivity is a compelling predictor of epidemic asthma in patients with grass pollen allergy and seasonal asthma. They focused on thunderstorm-related asthma which is most likely associated with external climatic factor [50]. Similarly, other studies [10, 15, 36, 37, 39] have recently reported a positive correlation of AE and tree pollen level, humidity and temperature dropping. Our results showed only a Mild, non-significant increase in number of AE in mold sensitized patients in the Season 1 (winter) than in Season 4 (33.63% vs. 28.32%, *p *> 0.05), when a higher count of Cladosporium was observed with max count in January: 14.623/m^3^ (not shown in the table). Cladosporium in general exceeds all other airborne biological particle in the atmosphere [45]. It is a predominant genus in temperate climate [13, 30], as well as in the region. Hasnain and collaborates in 2004 documented that Cladosporium consisted 25% of all spores, being the most abundant mold species in the Middle East region [23]. The obtained results in the present study showed positive correlation with AE in Season 1 only, which is characterised with a dropping in the air temperature (mean temperature was 13–19 °C) and an occurrence of a higher chance of rain. However, our results showed a rare sensitization to Cladosporium. Isolated mono-sensitization to that species is not found at all (Table 2). Because of that we speculated that other non-allergic factors (viral infections, weather parameters, air pollution, etc.) may contribute to the occurrence of AE in desert countries, both in allergic and non-allergic asthmatics.

Alternaria species is the best and most investigated outdoor mold associated with allergy [45]. It is the only species that may be independently associated and responsible for severe epidemic AE [9, 39] in temperate climate conditions, especially in the harvesting season and during thunderstorms. Alternaria is however less abundant in the Middle East if compared with other geographical regions [14, 21, 42]. On the other side, we found the higher rate of sensitization to Alternaria (16.41%) if compared with the rate of sensitization to Aspergillus (10.36%) and Cladosporium: (9.67%) (Table 1). Such results might be under the influence of more advanced characterization and standardization of allergenic extracts of Alternaria species.

The obtained results are, in contrast to the previous study, conducted in Kuwait [17] based on mold-specific IgE only, and showed predominance in sensitization of asthma patients to Aspergillus (21.3%) than to Alternaria (15.9%) and Cladosporium (14.6%). However, the methodology and design of that study was different.

In addition, Alternaria and Cladosporium, in contrast to Aspergillus, contain non-thermo-tolerant allergens. Their allergenic effects are directly related to airborne content of the spores [37] in any particular season. Aspergillus, as a thermo-tolerant mold that shows low concentrations throughout the year, may express persistent allergenic stimulus in the airways in the absence of direct airborne exposure [37].

Our obtained results support difficulties to clarify mold as being a direct trigger of AE in a desert environment. Furthermore, the highest number of AEs in our study was linked to Season 4 (highest concentration of pollen spores). Overwhelming sensitization to pollens might affect the assessment of sensitization in poly-sensitized patients. Even though pollen grains are too large to penetrate into the lower airways, AE has long been linked to pollen [59] regardless of non-respirable size of the whole pollen grains (≥ 10 μm in diameter), in contrast to respirable allergenic molds (2.5 to 10 μm in diameter). It has been documented that when highly allergenic birch trees are exposed to the moisture and wind, during flowering, then pollen spores can produce aerosols particles which contain small enough pollen allergens (up to 5 μm) [58], with the potential to induce an allergic inflammatory response and immunological reactivity in the lower airways [15, 16].

Although the previous studies [19], documented that “change in weather”, as a non-allergic trigger, is less involved in the occurrence of AE, meteorological parameters might exhibit a substantial influence on the concentration of atmospheric allergenic bioaerosol [44, 49]. Thus, some authors documented significant impact of inter and intra-day change in humidity 2 days before the admission date for asthma [30, 40]. Similarly, our previous study [54], showed a close relationship in the increased number of visits of symptomatic asthma patients with significant increase in relative humidity. The results in the present study support that phenomenon, showing significant correlation between AEs in mold-sensitized patients with relative humidity and atmospheric pressure (*p *= 0.022 and *p *= 0.028, respectively) (Table 4). In addition, the previous study done in Kuwait [51], observed fungal spore counts were significantly higher in early winter (December). The same authors found that the high fungal spore count might be related with high relative humidity, precipitation and the low mean average temperature of 19.7 °C. They concluded that the increased number of asthmatic patients visiting an emergency clinic during December was significantly associated with high aerial counts of fungal spores (*p *< 0.03).

In regard to the influence of atmospheric pressure, the obtained results in this study showed similar correlation in both groups (non-atopic *p *= 0.047 vs *atopic p *= 0034).

Arabian Peninsula is considered as one of the most difficult environments in the world to characterize, monitor and compare [53]. Moreover, Kuwait, with an area of 17,820 km^2^, has the highest surface temperature in the Arabian Peninsula [53, 54]. Seasonal variation of wind and dust over Kuwait may amplify pollens spread. It seems that weather parameters might influence atmospheric chemical reactions, affect atmospheric transport process [6] and affect both pollen production and pollen allergencity, Other authors showed that high temperature and sunshine duration might have a strong positive correlation with the spore counts and increase their allergen potency [33]. A recent report from Polish cities indicated that the seasonal atmospheric spores’ parameters were in over 40% determined by air temperature and wind velocity [21], while diurnal temperature variations may show up a similar effect [52]. Kim and collaborates observed that an increase in daily temperatures correlated with an increase in the emergency room department visits for asthma, especially in patients older than 65 years [35]. Contrary to that, others [65] have documented that higher temperatures were not associated with asthma hospital admissions. This is consistent with our results, showing the negative correlation between air temperature and AE in both atopic and non-atopic. During the extremely high outdoor air temperature in summer in the desert environment, there were very little spores, with a low number of AE too. Similarly, remarkable inverse correlation between the number of AE and the temperature and positive correlation with RH was observed in a previous study conducted in Kuwait [56] which also support our results.

In conclusion, outdoor molds might not have a significant role in the development of AE in the desert. The obtained results suggest a possible additional role of molds in triggering AEs in pollination peak. Meteorological factors typical for the desert (extremely high temperature during long summer, low average RH and mild variation in AP) also didn’t show a significant role in triggering AEs in molds sensitized patients.

It seems that in the industrially developing country, located in the unique desert climate, other non-allergenic factors such as typical climates with common dusty days and high levels of air pollutants [54] create poor air quality and facilitate the allergenic potency of outdoor allergens, affecting more pollen than mold allergens.

As our results did not confirm mold sensitization as an isolated cause of AEs, further studies are necessary to identify other asthma triggers in desert regions, which might create the bases for an environmental secondary prevention.

## Abbreviations

AE: asthma exacerbation
SPT: skin prick test
ATS: American Thoracic Society
ERS: European Respiratory Society
WAO: World Allergy Organization
RH: relative humidity
T: temperature
AP: atmospheric pressure
n: number
F: female
m: male

## References

[1] Al-Suwaini AS, Bahkali AH, Hasnain SM (2011). Airborne viable fungi in Riyadh and allergenic response of their extracts. Mycoses.

[2] Al-Dowaisan A, Fakim N, Khan MR, Arifhodzic N, Panicker R, Hanoon A (2004). Salsola pollen as a predominant cause of respiratory allergies in Kuwait. Ann Allergy Asthma Immunol.

[3] Arbes SJ, Gergen PJ, Elliott L, Zeldin DC (2005). Prevalences of positive skin test responses to 10 common allergens in the US population: results from the third National Health and Nutrition Examination Survey. J Allergy Clin Immunol..

[4] Arroyave WD, Rabito FA, Carlson JC, Sever ML, Lefante J (2016). Asthma severity, not asthma control, is worse in atopic compared with nonatopic adolescents with asthma. Ann Allergy Asthma Immunol.

[5] Bavbek S, Erkekol FO, Ceter T, Mungan D, Ozer F, Pinar M (2006). Sensitization to Alternaria and Cladosporium in patients with respiratory allergy and outdoor counts of mold spores in Ankara atmosphere, Turkey. J Asthma..

[6] Beggs PJ (2010). Adaptation to impacts of climate change on aeroallergens and allergic respiratory diseases. Int J Environ Res Public Health.

[7] Behbehani N, Arifhodzic N, Al-Mousawi M, Marafie S, Ashkanani L, Moussa M (2004). The seasonal variation in allergic rhinitis and its correlation with outdoor allergens in Kuwait. Int Arch Allergy Immunol.

[8] Burge HA, Rogers CA (2000). Outdoor allergens. Environ Health Perspect.

[9] Bush RK, Portneoy JM, Saxon A, Terr AI, Wood RA (2006). The medical effects of mold exposure. J Allergy Clin Immunol..

[10] Crameri R, Garbani M, Rhyner C, Huitema C (2014). Fungi: the neglected allergenic sources. Allergy.

[11] D’Amato G, Vitale C, D’Amato M, Cecchi L, Liccardi G, Molino A (2016). Thunderstorm-related asthma: what happens and why. Clin Exp Allergy.

[12] Dales RE, Cakmak S, Judek S, Dann T, Coates F, Brook JR (2003). The role of fungal spores in thunderstorm asthma. Chest.

[13] de Ana SG, Torres-Rodríguez JM, Ramírez EA, García SM, Belmonte-Soler J (2006). Seasonal distribution of Alternaria, Aspergillus, Cladosporium and Penicillium species isolated in homes of fungal allergic patients. J Investig Allergol Clin Immunol.

[14] Delfino RJ, Zeiger RS, Seltzer JM, Street DH, Matteucci RM, Anderson PR (1997). The effect of outdoor fungal spore concentrations on daily asthma severity. Environ Health Perspect.

[15] Denning DW, O’Driscoll BR, Hogaboam CM, Bowyer P, Niven RM (2006). The link between fungi and severe asthma: a summary of the evidence. Eur Respir J.

[16] Emberlin J (1995). Plant allergens on pauci-micronic airborne particles. Clin Exp Allergy.

[17] Ezeamuzie CI, Al-Ali S, Khan M, Hijazi Z, Dowaisan A, Thomson MS (2000). IgE-mediated sensitization to mould allergens among patients with allergic respiratory diseases in a desert environment. Int Arch Allergy Immunol.

[18] Fukutomi Y, Taniguc M (2015). Sensitization to fungal allergens: resolved and unresolved issues. Allergol Int..

[19] Gautier C, Charpin D (2017). Environmental triggers and avoidance in the management of asthma. J Asthma Allergy..

[20] Goronfolah L (2016). Aeroallergens, atopy and allergic rhinitis in the Middle East. Eur Ann Allergy Clin Immunol..

[21] Grinn-Gofron A, Strzelczak A, Stepalska D, Myszkowska D (2016). A 10-year study of Alternaria and Cladosporium in two Polish cities (Szczecin and Cracow) and relationship with the meteorological parameters. Aerobiologia (Bologna)..

[22] Harmanci E, Metintas M, Erginel S (2000). Respiratory allergy to moulds among adults in Eskisehir Anatolia, Turkey. Allerg Immunol (Paris)..

[23] Hasnain SM, Al-Frayh AS, Al-Suwaine A, Gad-El-Rab MO, Fatima K, Al-Sedairy S (2004). Cladosporium and respiratory allergy: diagnostic implications in Saudi Arabia. Mycopathologia.

[24] Hasnain SM, Al-Frayh AR, Subiza JL, Fernandez-Caldas E, Casanovas M, Geith T (2012). Sensitization to indigenous pollen and molds and other outdoor and indoor allergens in allergic patients from Saudi arabia, United arab emirates, and Sudan. World Allergy Organ J.

[25] Hawkes CV, Kivlin SN, Rocca JD, Huguet V, Thomsen MA, Suttle KB (2011). Fungal community responses to precipitation. Glob Change Biol.

[26] Heinzerling L, Frew AJ, Bindslev-Jensen C, Bonini S, Bousquet J, Bresciani M (2005). Standard skin prick testing and sensitization to inhalant allergens across Europe:a survey from the GALEN network. Allergy.

[27] Helbling A, Reimers A (2003). Immunotherapy in fungal allergy. Curr Allergy Asthma Rep..

[28] Horst M, Hejjaoui A, Horst V, Michel FB, Bousquet J (1990). Double-blind, placebo-controlled rush immunotherapy with standardized Alternaria extract. J Allergy Clin Immunol..

[29] Jara D, Portnoy J, Dhar M, Barnes C (2017). Relation of indoor and outdoor airborne fungal spore levels in the Kansas City metropolitan area. Allergy Asthma Proc..

[30] Kalyoncu F (2010). Relationship between airborne fungal allergens and meteorological factors in Manisa City, Turkey. Environ Monit Assess..

[31] Karvala K, Nordman H, Luukkonen R, Nykyri E, Lappalainen S, Hannu T (2008). Occupational rhinitis in damp and moldy workplaces. Am J Rhinol..

[32] Kennedy JL, Heymann PW, Platts-Mills TA (2012). The role of allergy in severe asthma. Clin Exp Allergy.

[33] Khwarahm N, Dash J, Atkinson PM, Newnham RM, Skjøth CA, Adams-Groom B (2014). Exploring the spatio-temporal relationship between two key aeroallergens and meteorological variables in the United Kingdom. Int J Biometeorol.

[34] Kilic M, Altintas DU, Yilmaz Kendirli SG M, Karacoc GB, Taskin E (2010). The effects of meteorological factors and Alternaria spore concentrations on children sensitised to Alternaria. Allergol Immunopathol.

[35] Kim J, Lim Y, Kim H (2014). Outdoor temperature changes and emergency department visits for asthma in Seoul, Korea: a time-series study. Environ Res.

[36] Kolodziejczyk K, Bozek A (2016). Clinical distinctness of allergic rhinitis in patients with allergy to molds. Biomed Res Int.

[37] Lombardi C, Savi E, Ridolo E, Passalacqua G, Canonica GW (2017). Is allergic sensitization relevant in severe asthma? Which allergens may be culprit?. World Allergy Organ J..

[38] Loymans RJ, Ter Riet G, Sterk PJ (2011). Definitions of asthma exacerbations. Curr Opin Allergy Clin Immunol.

[39] May L, Carim M, Yadav K (2011). Adult AE and environmental triggers: a retrospective review of ED visits using an electronic medical record. Am J Emerg Med.

[40] Mireku N, Wang Y, Ager J, Reddy RC, Baptist AP (2009). Changes in weather and the effects on pediatric AE. Ann Allergy Asthma Immunol.

[41] Moghtaderi M, Hosseini Teshnizi S, Farjadian S (2017). Sensitization to common allergens among patients with allergies in major Iranian cities: a systematic review and meta-analysis. Epidemiol Health..

[42] Newson R, Strachan D, Corden J, Millington W (2000). Fungal and other spore counts as predictors of admissions for asthma in the Trent region. Occup Environ Med.

[43] O’Driscoll BR, Hopkinson LC, Denning DW (2005). Mold sensitization is common amongst patients with severe asthma requiring multiple hospital admissions. BMC Pulm Med..

[44] Oliveira M, Ribeiro H, DelgadoI JL, Abreu I (2009). The effects of meteorological factors on airborne fungal spore concentration in two areas differing in urbanisation level. Int J Biometeorol.

[45] Ozdemir O (2015). Molds and respiratory allergy—part 1. MOJ Immunol.

[46] Pongracic JA, O’Connor GT, Muilenberg ML, Vaughn B, Gold DR, Kattan M (2010). Differential effects of outdoor versus indoor fungal spores on asthma morbidity in inner-city children. J Allergy Clin Immunol..

[47] Portnoy JM, Barnes CS, Kennedy K (2008). Importance of mold allergy in asthma. Curr Allergy Asthma Rep.

[48] Portnoy JM, Jara D (2015). Mold allergy revisited. Ann Allergy Asthma Immunol.

[49] Priyamvada H, Singh RK, Akila M, Ravikrishna R, Verma RS, Gunthe SS (2017). Seasonal variation of the dominant allergenic fungal aerosols—One year study from southern Indian region. Sci Rep..

[50] Pulimood TB, Corden JM, Bryden C, Sharples L, Nasser SM (2007). Epidemic asthma and the role of the fungal mold Alternaria alternate. J Allergy Clin Immunol..

[51] Qasem JA, Nasrallah H, Al-Khalaf BN, Al-Sharifi F, Al-Sherayfee A, Almathkouri SA (2008). Meteorological factors, aeroallergens and asthma-related visits in Kuwait: a 12-month retrospective study. Ann Saudi Med.

[52] Qiu H, Yu IT, Tse LA, Chan EY, Wong TW, Tian L (2015). Greater temperature variation within a day associated with increased emergency hospital admissions for asthma. Sci Total Environ.

[53] Sabbah I, Al-Mudhaf HF, Al Kandari A, Al-Sharifi F (2012). Remote sensing of desert dust over Kuwait: long-term variation. Atmos Pollut Res.

[54] Sabbah I, Arifhodzic N, Al-Ahmad M, Ali-Enizi A, Al-Haddad A, Al-Ajmi N (2014). Influence of air quality conditions on asthmatic patient visits in Kuwait. J Allergy Ther..

[55] Simon-Nobbe B, Denk U, Pöll V, Rid R, Breitenbach M (2008). The spectrum of fungal allergy. Int Arch Allergy Immunol.

[56] Strannegård IL, Strannegård O (1987). Asthma and serum IgE levels in children in a desert country. Int Archs Allergy Appl Immunol.

[57] Sttenet PJ, Beggs PJ (2004). Alternaria spores in the atmosphere of Sydney, Australia, and relationships with meteorological factors. Int J Biometeorol..

[58] Taylor PE, Flagan RC, Miguel AG, Valenta R, Glovsky MM (2004). Birch pollen rupture and the release of aerosols of respirable allergens. Clin Exp Allergy.

[59] Taylor PE, Flagan RC, Valenta R, Glovsky MM (2002). Release of allergens as respirable aerosols: a link between grass pollen and asthma. J Allergy Clin Immunol..

[60] Tham R, Vicendese D, Dharmage SC, Hyndman RJ, Newbigin E, Lewis E (2017). Associations between outdoor fungal spores and childhood and adolescent asthma hospitalizations. J Allergy Clin Immunol.

[61] Torres-Rodriguez JM, Pulido-Marrero Z, Vera-Garcia Y (2012). Respiratory allergy to fungi in Barcelona, Spain: clinical aspects, diagnosis and specific treatment in a general allergy unit. Allergol Immunopathol (Madr)..

[62] Twaroch TE, Curin M, Valenta R, Swoboda I (2015). Mold allergens in respiratory allergy: from structure to therapy. Allergy Asthma Immunol Res..

[63] Yamamoto N, Bibby K, Qian J, Hospodsky D, Rismani-Yazdi H, Nazaroff WW (2012). Particle-size distributions and seasonal diversity of allergenic and pathogenic fungi in outdoor air. ISME J.

[64] Zeldin Y, Kidon MI, Magen E, Bibi H, Cohen A, Waisel Y (2008). Impact of specific allergen sensitization on the prevalence of asthma in patients with allergic rhinitis from adjacent distinct geographic areas. Ann Allergy Asthma Immunol.

[65] Zhang Y, Peng L, Kan H, Xu J, Chen R, Liu Y (2014). Effects of meteorological factors on daily hospital admissions for asthma in adults: a time-series analysis. PLoS ONE.

[66] Żukiewicz-Sobczak W, Krasowska E, Zwoliński J, Sobczak P, Chmielewska-Badora J, Wróblewska P (2012). Allergic diseases—current state of knowledge. Postep Derm Alergol..

[67] Zureik M, Neukirch C, Leynaert B, Liard R, Bousquet J, Neukirch F, European Community Respiratory Health Survey (2002). Sensitisation to airborne moulds and severity of asthma: cross sectional study from European Community respiratory health survey. BMJ.

